# Evolution of Susceptibility to Ingested Double-Stranded RNAs in *Caenorhabditis* Nematodes

**DOI:** 10.1371/journal.pone.0029811

**Published:** 2012-01-11

**Authors:** Isabelle Nuez, Marie-Anne Félix

**Affiliations:** Institut Jacques Monod, Centre National de la Recherche Scientifique - Université Paris Diderot, Paris, France; Centre National de la Recherche Scientique and University of Nice Sophia-Antipolis, France

## Abstract

**Background:**

The nematode *Caenorhabditis elegans* is able to take up external double-stranded RNAs (dsRNAs) and mount an RNA interference response, leading to the inactivation of specific gene expression. The uptake of ingested dsRNAs into intestinal cells has been shown to require the SID-2 transmembrane protein in *C. elegans*. By contrast, *C. briggsae* was shown to be naturally insensitive to ingested dsRNAs, yet could be rendered sensitive by transgenesis with the *C. elegans sid-2* gene. Here we aimed to elucidate the evolution of the susceptibility to external RNAi in the *Caenorhabditis* genus.

**Principal Findings:**

We study the sensitivity of many new species of Caenorhabditis to ingested dsRNAs matching a conserved actin gene sequence from the nematode Oscheius tipulae. We find ample variation in the Caenorhabditis genus in the ability to mount an RNAi response. We map this sensitivity onto a phylogenetic tree, and show that sensitivity or insensitivity have evolved convergently several times. We uncover several evolutionary losses in sensitivity, which may have occurred through distinct mechanisms. We could render C. remanei and C. briggsae sensitive to ingested dsRNAs by transgenesis of the Cel-sid-2 gene. We thus provide tools for RNA interference studies in these species. We also show that transgenesis by injection is possible in many Caenorhabditis species.

**Conclusions:**

The ability of animals to take up dsRNAs or to respond to them by gene inactivation is under rapid evolution in the *Caenorhabditis* genus. This study provides a framework and tools to use RNA interference and transgenesis in various *Caenorhabditis* species for further comparative and evolutionary studies.

## Introduction

RNA interference is the inactivation of gene expression induced by double-stranded RNAs (dsRNAs) [1]. The molecular mechanism by which dsRNAs introduced into a cell induce degradation of the corresponding mRNA has been well documented in the nematode *Caenorhabditis elegans*. In this species, dsRNAs do not need to be injected, but are able to enter cells when the animal is either soaked in a dsRNA preparation [2], or is fed *E. coli* bacteria that express dsRNAs from a plasmid [3].

In a screen for *C. elegans* mutants that are specifically defective in the uptake of externally administered dsRNAs, but not in the downstream response (Sid phenotype, for systemic-interference-defective), Winston et al. [4] found mutants at two loci, named *sid-1* and *sid-2*. The *sid-1* gene encodes a transmembrane protein that is necessary for spread of the RNAi signal within the organism [4], whereas the *sid-2* gene is required for the primary uptake of dsRNAs into intestinal cells [5]. Interestingly, *C. briggsae*, a close relative of *C. elegans*, encodes a divergent form of *sid-2*, which appears unable to take up dsRNAs. The inability of *C. briggsae* to take up ingested dsRNAs can be rescued by expression of the *C. elegans sid-2* (*Cel-sid-2*) gene in *C. briggsae* [5].

Winston et al. [5] tested other *Caenorhabditis* species available at this time for sensitivity to external dsRNAs, by soaking each of them in species-specific dsRNAs matching the large subunit of RNA polymerase II (*ama-1*) gene. Except for *Caenorhabditis* sp. 1 SB341 (the species diverging most basally), other *Caenorhabditis* species, including *C. briggsae*, *C. remanei* and *C. brenneri*, were found incapable of mounting an RNAi response upon external dsRNA application (soaking) [5]. Outside the *Caenorhabditis* genus, many nematode species were also found to be incapable of mounting an RNAi response [6], [7], but some are sensitive, even to ingested dsRNAs [8], [9]. These results highlight that the choice to develop C. elegans as a model organism was fortunate given its RNAi sensitivity [9]. The results further raise questions of how selective forces have shaped the evolutionary divergence of the RNAi machinery, including the import of dsRNAs and the *sid-2* gene. Using artificial and natural viruses, the RNAi machinery has been shown to be involved in anti-viral responses [10], [11], [12], [13]. Genes involved in immune responses, and specifically, those involved in the RNAi response evolve rapidly, in *Drosophila melanogaster* [14] as well as in *C. elegans* (Supplement in [15]) and other nematodes [5], [16]. This raises the possibility that the RNAi response is evolutionarily very labile.

In recent years, many new *Caenorhabditis* species have been discovered, brought in laboratory culture and their phylogenetic relationships determined [17]. With the practical goal of evaluating the possibility of using external dsRNA application for gene inactivation and transgenesis in these new *Caenorhabditis* species, we here investigate their ability to respond to ingested dsRNAs, using a conserved actin gene fragment from an outgroup species, *Oscheius tipulae*. We map the external RNAi competency data onto the phylogenetic tree of the genus and deduce possible evolutionary patterns for this RNAi response. Using *Cel-sid-2* transgenesis, we produced *C. remanei* and *C. briggsae* lines that can be used for a wide range of RNAi experiments using ingested dsRNAs. Finally, in a wide range of *Caenorhabditis* species we tested transgenesis and the usefulness of several transformation markers.

## Methods

### Strains

- Wild type isolates tested for RNAi: *Caenorhabditis elegans* N2, CB4856, JU1580, *C. briggsae* AF16, JU1264 and HK104 (data not shown, Amélie Broucke), *C. remanei* PB4641, *C. brenneri* PB2801, *C. drosophilae* DF5077, *C. plicata* SB355, *C.* sp. 2 DF5070, *C. angaria* RGD1, *C.* sp. 5 JU727, *C. s*p. 6 EG4788 (gift of M. Ailion), *C.* sp. 7 JU1199 and JU1593, *C.* sp. 8 QX1182 (gift of M. Rockman), *C.* sp. 9 JU1325, *C.* sp. 10 JU1333, *C.* sp. 11 JU1373 (V. Robert and M.-A. Félix), *C.* sp. 12 JU1427 (P. Châtelet and M.-A. Félix), *C.* sp. 13 JU1528, *C.* sp. 14 EG5716 (gift of M. Ailion), *C.* sp. 15 QG122 (gift of M. Rockman), *C.* sp. 16 JU1873 (J.-B. Pénigault), *C.* sp. 17 JU1825 (C. Braendle and M.-A. Félix), *C.* sp. 18 JU1857 (C. Braendle and M.-A. Félix), *C.* sp. 19 EG6142 (gift of M. Ailion), C. sp. 20 NIC113 (gift of C. Braendle)

- Additional wild type isolates tested for transformation: *C. remanei* SB146, *C. brenneri* CB5161, *C. angaria* PS1010; *C. plicata* SB355, *C.* sp. 1 SB341 (gift of W. Sudhaus).

- Transgenic strains (see construction below): *C. briggsae* JU1018 (*mfIs42[Cel-sid-2; Cel-myo-2::DsRed2]*); JU1076 (*mfIs48[Cel-sid-2; Cel-myo-2::DsRed2]*); *C. remanei* JU1184 (*mfEx34[Cel-sid-2; Cel-myo-2::DsRed2]*, likely integrated); *C.* sp. 9 JU1591 (*mfEx65[Cel-sid-2; Cel-myo-2::DsRed2]*); *C.* sp. 11 JU1592 (*mfEx66[Cel-sid-2; Cel-myo-2::DsRed2]*) and JU1874 (*mfEx44[Cel-sid-2; Cel-myo-2::GFP]*).

### Generation of dsRNA-expressing bacteria

*- Oti-actin* RNAi plasmid: An *Oti-actin* fragment was isolated using primers designed using the *C. elegans* actin sequence: primers actinI (ATGTGCAAGGCCGGATTCG) and actinIII (ATAGGGACGGTGTGG). The PCR was performed on genomic DNA of the *Oscheius tipulae* CEW1 strain with an annealing temperature of 55°C and 2.5 mM MgCl_2_ [18]. The amplified fragment was inserted into the pGEMTeasy plasmid, yielding pMD13. After digestion of pMD13 by NotI, the *Oti-actin* fragment was inserted into pPD129.36 [3] and the resulting pMA33 plasmid transformed into chemically competent HT115 cells used for RNAi experiments. The sequence of the *Oti-actin* fragment amplified with actinI and actinIII is:

“ATGTGCAAGGCCGGATTCGCCGGAGACGATGCTCCTCGCGCCGTCTTCCCCTCCATCGTCGGTCGCCCCCGTCACCAGGGAGTCATGGTCGGAATGGGACAGAAGGACAGCTACGTCGGAGACGAGGCCCAGTCCAAGAGAGGTATCCTGACCCTCAAGTACCCCATTGAGCACGGTATCGTCACCAACTGGGATGACATGGAGAAGATCTGGCACCACACCTTCTACAATGAGCTCCGTGTCGCCCCTGAGGAGCACCCCGTCCTCCTGACTGAGGCTCCCCTCAATCCCAAGGCTAACCGTGAGAAGATGACCCAGATCATGTTCGAGACCTTCAACGCTCCCGCCATGTACGTCGCCATCCAGGCCGTCCTCTCCCTCTACGCCTCCGGACGTACCACCGAGTCGTCCTCGACTCTGGAGATGGAGTTACCCACACCGTCCCTAT”.

The matching actin gene sequences of *C. elegans*, *C. briggsae*, *C. remanei*, *C. brenneri* and *C. angaria* are available in Wormbase (www.wormbase.org). Those of the other species have not been determined.

- *Cre-unc-22* RNAi plasmid: A *Cre-unc-22* fragment was amplified using primers oMA197 (AGGCGGCCGCGTCGAAGAAGACGACCAAGC) containing a NotI restriction site and oMA198 (TGCCATGGTCCAGCTTCGCTTCAATTTT) containing a NcoI restriction site. This PCR fragment was digested with NcoI and NotI, and inserted into pPD129.36 plasmid, previously digested with NcoI and NotI. Genomic sequences of *Caenorhabditis* species can be found in Wormbase (www.wormbase.org).

*- Can-ama-1* and *Cdr-ama-1* RNAi plasmids: A *Can-ama-1* fragment was amplified from *C. angaria* genomic DNA using primers oIN114 (GGGGACAAGTTTGTACAAAAAAGCAGGCTCCCTACACTGAAGATGTGATC) and oIN115 (GGGGACCACTTTGTACAAGAAAGCTGGGTGTGTTGTATGCTCCAATTTGC). A *Cdr-ama-1* fragment was amplified from *C. drosophilae* genomic DNA using primers oIN124 (GGGGACAAGTTTGTACAAAAAAGCAGGCTGCGATGGGAGGTCGTGAAG) and oIN125 (GGGGACCACTTTGTACAAGAAAGCTGGGTCGAATCACATCTTCCGAG). oIN114 and oIN124 contain an attB1 forward site, while oIN115 and oIN125 contain an attB2 reverse site. Fragments oIN124-oIN125 and oIN114-oIN115 were inserted into pDONR221 according to BP reaction's protocol (Multisite Gateway Invitrogen) and a LR reaction was performed to insert the *ama-1* fragment into the pDEST L4440 Gateway feeding vector. Chemically competent HT115 cells were transformed with these Gateway RNAi *ama-1* feeding plasmids. *ama-1* codes for RNA polymerase II and is called *pol-2* in [5]. We use here the Wormbase name.

- *Cbr-lin-12* RNAi plasmid: A fragment of *Cbr-lin-12* gene was amplified with oMA158 (AGccatggTACTTGTTCTTC) and oMA159 (TGgcggccgcTTCTCACTGAACATT). This PCR fragment was digested with NcoI and NotI, and inserted into the pPD129.36 plasmid previously digested with NcoI and NotI, yielding pMA52.

- *Cel-rol-6*, *Cel-pos-1* and *Cel-unc-22* RNAi clones were from the Ahringer library [19].

### dsRNA feeding protocol

Bacteria were grown overnight in LB with 20 µg/mL ampicillin and 12.5 µg/mL tetracyclin. They were then seeded onto 55 mm diameter NGM agar plates containing 1 mM IPTG, 20 µg/mL ampicillin and 12.5 µg/mL tetracyclin. Seeded plates were incubated at room temperature for 2 days.

For hermaphroditic species, two RNAi plates with three L4-stage hermaphrodites were incubated at 23°C for 24 hrs. Then, for each plate, each animal was isolated onto a new RNAi plate seeded with the same bacteria, allowed to lay eggs overnight (ca. 16 hrs) at 23°C and then removed. Its progeny was scored after 24 hrs at 23°C for phenotypes related to *actin* RNAi and 48 hrs for phenotypes related to *unc-22* RNAi.

For male-female species, eight L4-stage females and four males were incubated at 23°C for 24 hrs on NGM plates seeded with HT115 bacteria expressing dsRNAs for the gene of interest. Then, six fertilized females were isolated onto a new RNAi plate and allowed to lay eggs for 16 hrs at 23°C before being removed.

For *Oti-actin* RNAi experiments, the progeny was scored two days later with a stereomicroscope for number of wild-type larvae, number of deformed larvae (L1s and later stages), and number of dead embryos. For *Cel-pos-1* and *ama-1*, the progeny was scored for number of wild-type larvae and number of dead embryos. For *unc-22*, we scored three days later the twitching phenotype of the adult progeny.

### Generation of dsRNAs for injections

PCR products corresponding to a part of the *Oti-actin* or *Cel-unc-22* genes were generated with T7 primer 5′-AATACGACTCACTATAG-3′ [20] from the *Oti*-*actin* or *Cel-unc-22* RNAi clones, using the following cycle conditions: 95°C 50 sec, 52°C 30 sec, 72°C 90 sec for 25 cycles. Purification of the PCR product was performed with Nucleospin Extract II kit (Macherey Nagel). *Oti-actin* or *Cel-unc-22* dsRNAs were then generated using the MEGAscript kit (Ambion).

dsRNAs were injected at 50 ng/µL into both gonadal arms of wild-type young adult hermaphrodites or females - depending on the reproductive mode of the species. For hermaphroditic species, the injected animal was transferred to a fresh plate 24 hrs after injection, and cultured at 23°C. For male-female species, a male was added onto the plate with the injected female and transferred with the female the day after. Phenotypes were scored as described above.

### Generation of *Cel-sid-2* transgenic animals

A *Cel-sid-2* PCR product was obtained from N2 genomic DNA using primers oMA183 (GCTCAAAACCAACCTTAACTGC) and oMA184 (TCTTGCATGGTCCCCAAGTA). This product was injected into *C. briggsae* AF16, *C. remanei* PB4641 and *C.* sp. 11 JU1373 at a concentration of 15 ng/µL, mixed with pWD47 (*Cel-myo-2::DsRed2*) at 10 ng/µL and carrier pBS to a final concentration of 150 ng/µL.

*C. briggsae* JU1018 and JU1076 are integrated lines produced by γ-irradiation of the transgenic strain JU977 (*mfEx32[Cel-sid-2; Cel-myo-2::DsRed2]*), backcrossed five times to AF16.

### Transgenesis in different *Caenorhabditis* species

The syncytial female germ line of different *Caenorhabditis* species was injected using the same protocol as in *C. elegans* [21], [22], injecting both gonadal arms when possible. Each injected animal was then transferred singly to a culture plate (except in the *C. plicata* experiment where two females were placed on each plate to reduce plate consumption). For male-female species, males were added to the plate with the injected female. The marker phenotype was assayed in the F1 and F2 progeny of each injected parent. The proportion of injected parents that show positive F1 or F2 progeny, respectively, is indicated in Table 1.

**Table 1:** Transgenesis of *Caenorhabditis* species.

Proportion of injected P0 animals yielding:	transgenic F1	transgenic F2
**(A) ** ***Cel-hsp-16-48::GFP*** ** pJL53 injected at 100 ng/µl**		
*C. elegans* N2	8/8	2/8
*C. briggsae* AF16	12/12	4/12
*C. remanei* SB146	2/11	0/11
*C. brenneri* CB5161	0/9	0/9
*C. angaria* PS1010	0/10	0/10
**(B) ** ***Cel-hsp-16-48::GFP*** ** pJL53 injected at 700 ng/µl**		
*C. elegans* N2	12/12	6/12
*C. remanei* SB146	13/15	0/15
*C. brenneri* CB5161	0/8#	-
*C. angaria* PS1010	0/9#	-
**(C) ** ***rol-6*** ** dominant pRF4 injected at 100 ng/µl**		
*C. elegans* N2	9/10	nd
*C. briggsae* AF16	9/10	nd
*C. remanei* SB146	7/12	0/12
*C. brenneri* CB5161	14/18	0/18
*C. angaria* PS1010	≥3/11	0/11
**(D) ** ***rol-6*** ** pRF4 100 ng/µl+** ***hsp-16-48::GFP*** ** pJL53 100 ng/µl**		
*C. elegans* N2	6/6	nd
*C. brenneri* CB5161	8/8#	nd
*C. angaria* PS1010	7/8#	nd
**(E) ** ***rol-6*** ** pRF4 100 ng/µl+** ***sur-5::GFP*** ** pTG96 100 ng/µl**		
*C. elegans* N2	7/7	7/7
*C. briggsae* AF16	10/11	8/11
*C. brenneri* CB5161	9/11	1/11 (100% transmission)
**(F) ** ***Cel-sur-5::GFP*** ** pTG96 100 ng/µl**		
*C. brenneri* CB5161	15/18	1/18
*C. angaria* PS1010	16/24	3/24
**(G) ** ***Cel-sur-5::GFP*** ** pTG96 150 ng/µl**		
*C.* sp. 2 DF5070	9/27	2/27
*C. plicata* SB355	4/9	0/18
*C.* sp. 1 SB341	0/51	-
**(H) ** ***Cel-myo-2::GFP*** ** pPD118.33 ng/µl**+*Cbr-egl-17::GFP* 150 ng/µl		
*C. briggsae* AF16	nd	4/10
*C. remanei* PB4641	nd	1/25
**(I) ** ***Cel-myo-2::GFP*** ** pPD118.33 5 ng/µl**+*daf-6::GFP* 50 ng/µl+*egl-17::CFP* 50 ng/µl		
*C. brenneri* CB5161	nd	2/21 (1 with 100% transmission)
**(J) ** ***Cel-myo-2::GFP*** ** pPD118.33 50 ng/µl**+*lin-3(+)* pRH9 100 ng/µl		
*C. angaria* RGD1 (day 1)	16/20	0/16
*C. angaria* RGD1 (day 2)	nd	2/20
*C.* sp. 2 DF5070	+	0/36
**(K) ** ***Cel-myo-2::GFP*** ** pPD118.33 50 ng/µl**+*daf-6::GFP* 100 ng/µl		
*C.* sp. 1 SB341	0/53	-

Transgenes that provide transgenesis markers are in bold.

# GFP marker is not expressed. “nd”: not determined.

The pJL53 plasmid (*Cel-hsp-16-18::GFP*) is a gift of Jean-Louis Bessereau (see [23] for the corresponding cis-regulatory sequences). To assay its expression, animals were heat-shocked at 33–37°C for 1.5–2 hrs and allowed to recover at 20°C for 1–2 hrs before scoring GFP fluorescence. The pRF4 plasmid contains a gain-of-function version of the *rol-6* collagen gene, *rol-6(su1006)*, which causes a dominant Roller phenotype [21]. The pTG96 (*Cel-sur-5::GFP*) plasmid is a gift from Min Han's laboratory and is expressed in most somatic cells [24]. The pPD118.33 (*Cel-myo-2::GFP*) plasmid is a gift from Andy Fire's laboratory and is expressed in the pharynx [25], [26]. The *Cel-myo-2::DsRed2* pWD47 plasmid is a gift of Wayne Davis and Erik Jorgensen.

### Statistical analyses

All statistical tests were performed with R (http://www.r-project.org/). A Mann-Whitney-Wilcoxon test was used to compare the numbers of wild-type larvae in the actin and *ama-1* RNAi experiments. An exact Fisher test was used on *Cre-unc-22* RNAi experimental results.

## Results

### Devising an RNAi test using a highly conserved gene

RNA interference requires a high degree of sequence similarity between the administered dsRNAs and the target gene. We looked for a dsRNA sequence that we could use in all *Caenorhabditis* species, and beyond the genus. Actin was a good candidate, being a highly conserved, abundant and essential cytoskeletal protein. Indeed, the actin genes are highly conserved at the nucleotide level, presumably because of their high level of expression, resulting in a strong codon usage bias, i.e. the nucleotides at synonymous positions tend to be conserved among closely related species and gene copies. In order to reduce a possible bias in efficiency of RNAi among *Caenorhabditis* species, we chose to score their respective sensitivity using an actin gene of a species outside the genus, namely *Oscheius tipulae* CEW1 [27] (see [28] for its phylogenetic position). We cloned an *Oti-actin* gene fragment in a plasmid containing two inducible T7 promoters (pPD129.36) that flank the gene fragment in mirror orientation.

**Figure 1:**
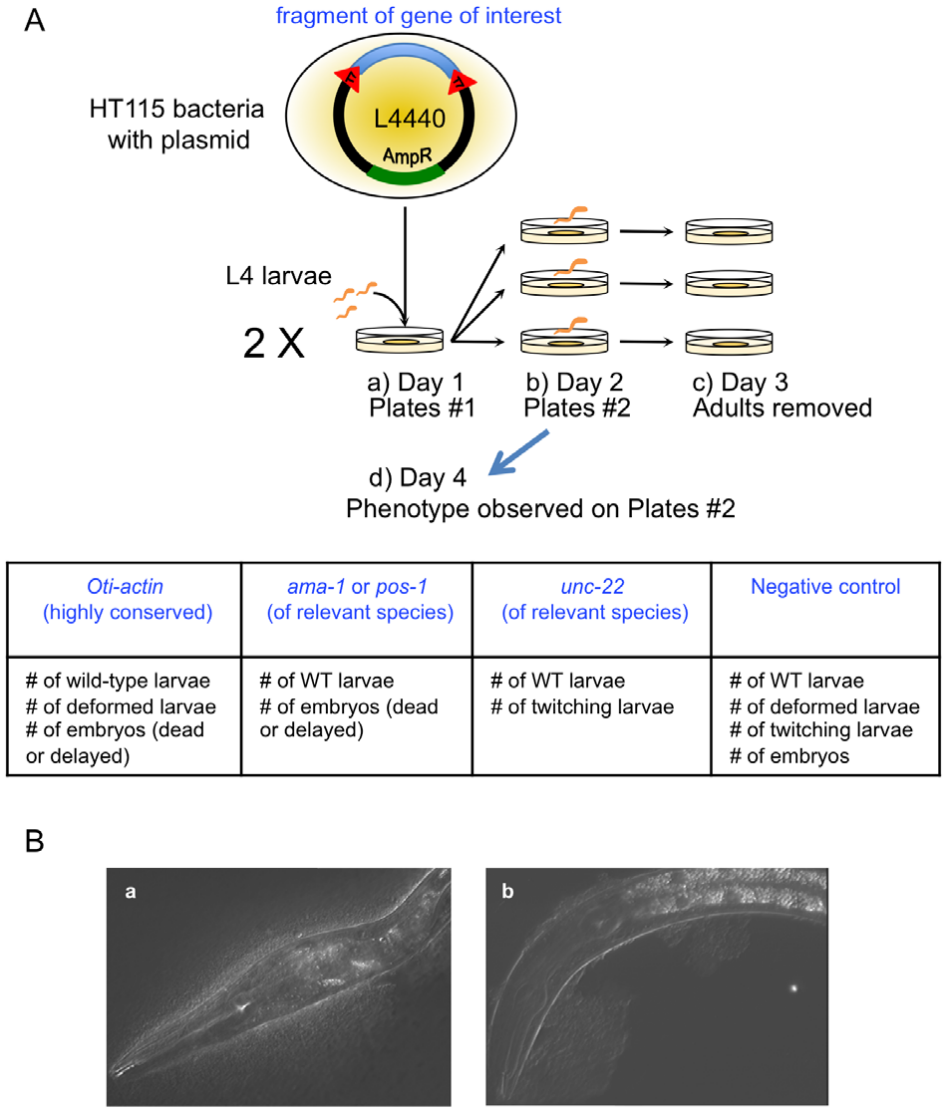
Outline of the RNAi test. (**A**) Experimental design of the RNAi test. a) L4 larvae were transferred on day 1 onto two RNAi plates seeded with bacteria producing *Oti-actin* dsRNAs. b) On day 2 (24 hrs later), the animals were isolated onto a new RNAi plate seeded with the same bacteria. c) On day 3 (after 16 hrs), the adults were removed. d) On day 4, the progeny was scored for the number of wild-type larvae, deformed larvae and embryos (these embryos must be highly delayed, arrested or dead). Experiments were performed at 23°C. (**B**) Morphological phenotype in the actin RNAi experiments. a) Deformed L1 larva of the *C. elegans* N2 strain on HT115 bacteria expressing *Oti-actin* dsRNAs (visualized here using Nomarski microscopy but visible under a dissecting microscope). b) Control L1 larva of the *C. elegans* N2 strain grown on control HT115 bacteria expressing *Cbr-lin-12* dsRNAs.

Inactivation of actin gene expression in *C. elegans* [29] results in a spectrum of phenotypes that appear during the course of the experiments, as a function of time and severity of defect. The mildest effect concerns deformation of the body of juveniles and adults grown on bacteria expressing *Oti-actin* dsRNAs (Figure 1), followed in order of increasing severity by a delayed growth of the juveniles, an embryonic arrest and finally sterility of the mother as the strongest and latest phenotype.

We devised a test of *Oti-actin* RNAi efficiency using these different phenotypic effects (Figure 1). We first incubated young adult animals for one day on dsRNA-expressing bacteria, transferred them to a new plate, allowed them to lay for a further 16 hours at 23°C and removed them. We then scored the progeny over the next days for the following traits: number of arrested embryos, number of deformed larvae, number of normal larvae. Most importantly, the number of progeny represents the fertility level of the mother. As a negative control, we used a clone of bacteria expressing dsRNAs that were not recognized by the tested species (e.g. *Cel-rol-6* or *Cbr-lin-12* when appropriate). In addition, *C. elegans* N2 was used as an internal positive control for each experiment.

### Evolutionary variation in sensitivity to external *Oti-actin* dsRNAs in the *Caenorhabditis* genus

**Figure 2:**
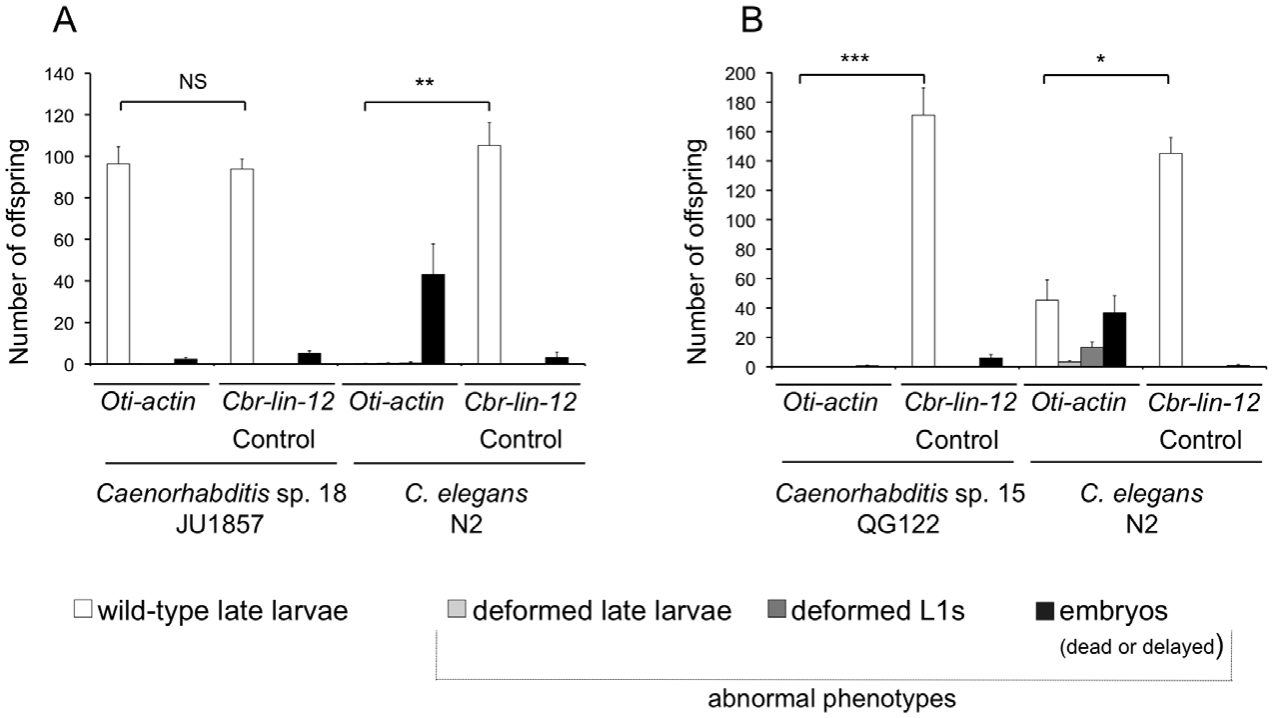
Comparison of sensitivity to ingested *Oti-actin* dsRNAs of *C. elegans* N2 to two examplary species. (**A**) *C.* sp. 18 JU1857 is insensitive to RNAi by feeding, whereas *C. elegans* N2 shows a response. *C.* sp. 18 JU1857 and *C. elegans* N2 progeny were scored using the experimental design described in Figure 1, using *Oti-actin* and *Cbr-lin-12* (negative control). (**B**) *C.* sp. 15 QG122 is highly sensitive to ingested *Oti-actin* dsRNAs. Here, feeding of *Oti-actin* dsRNA resulted in complete sterility of the mothers, whereas in the same experiment, treated N2 mothers produced arrested embryos and larvae. Statistical comparisons were made to the results of control experiments with *Cbr-lin-12* dsRNA, which has no effect on either strain, using a Mann-Whitney-Wilcoxon rank sum test on the number of normal larval progeny of each parent. The significance of the difference is depicted as follows: (**NS**) non-significant, (*) 0.01<*p*<0.05, (**) 0.001<*p*<0.01, (***) *p*<0.001. Error bars indicate the standard error of the mean over individuals (n = 6–12, see Table S1 for details).

**Figure 3:**
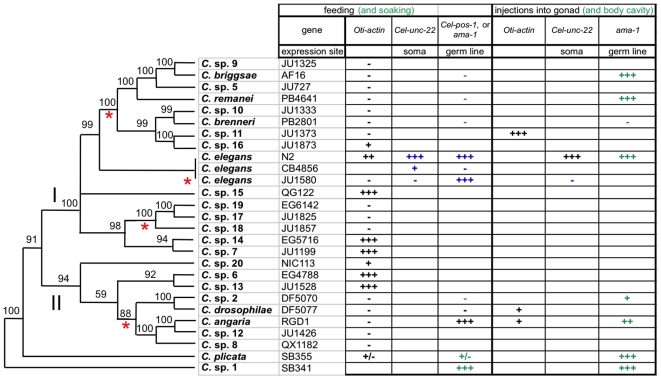
Evolution of RNAi sensitivity in the *Caenorhabditis* genus. The RNAi results are displayed on the phylogeny from [17], for the different dsRNA administration methods and for different genes. The red stars indicate a loss of sensitivity to ingested *Oti-actin* dsRNAs in a parsimonious evolutionary scenario (see text). Note that the absence of an external *Oti-actin* RNAi response may correspond to an inability to respond either to all ingested dsRNAs (as in *C. briggsae* and likely in *C. drosophilae*), or only to specific dsRNAs (as in *C. elegans* JU1580 and *C. angaria*). In blue: results from [13]. In green: result̀s from [5]. The dsRNAs for the *ama-1* gene were specific to the tested species. We did not find differences among different tested isolates of *C. briggsae* and *C.* sp. 7 (see Materials and Methods for the identity of other tested strains), nor did Winston et al. for different isolates of *C. brenneri* [5]. *C. brenneri* can respond to *ama-1* dsRNAs if they are injected into the gonad, indicating that this species is defective in the systemic spread of the signal from body cavity to gonad. I: *Elegans* supergroup of *Caenorhabditis* species; II: *Drosophilae* supergroup as in [17].

We systematically assayed all species of *Caenorhabditis* in culture for their sensitivity to external application of dsRNA (except for *C. japonica* strains, which have a very poor fecundity [5]; our observations). We first confirmed in our assay previous results that had been obtained by soaking the animals in dsRNAs corresponding to the species *ama-1/pol-2* (coding for the RNA polymerase II large subunit) gene [5]. We further scored many new species, some of which were found to be naturally sensitive to external dsRNAs. Figure 2 shows examples of a species that is insensitive to ingested *Oti-actin* dsRNAs, *Caenorhabditis* sp. 18 (Figure 2A), and of a highly sensitive species, *Caenorhabditis* sp. 15 (Figure 2B). Further results are reported in Table S1 and Figure 3.

In addition to *C. elegans* and *C.* sp. 1 SB341, we found that *Caenorhabditis* sp. 6 EG4788, *C.* sp. 7 JU1199, *C.* sp. 13 JU1528, *C.* sp. 14 EG5716 and *C.* sp. 15 QG122 were naturally sensitive to ingested dsRNAs and thus easily amenable to RNAi screens. *C.* sp. 16 JU1873, *C.* sp. 20 NIC113 and perhaps *C. plicata* appeared mildly sensitive to ingested *Oti-actin* dsRNAs. In contrast, *C. briggsae*, *C. remanei*, *C. brenneri*, *C. drosophilae*, *C.* sp. 2 DF5070, *C.* sp. 3 RGD1, *C.* sp. 5 JU727, *C.* sp. 8 QX1182, *C.* sp. 9 JU1325, *C.* sp. 10 JU1333, *C.* sp. 11 JU1373, *C.* sp. 12 JU1428, *C.* sp. 17 JU1825, *C.* sp. 18 JU1857 and *C.* sp. 19 EG6142 were insensitive. Insensitivity to ingested *Oti-actin* dsRNAs is thus predominant in terms of number of species. Importantly, it does not follow a phylogenetic distribution (see below) that would suggest that it can be explained by the sequence divergence of the actin gene of the species relative to the *Oti-actin* gene fragment.

It is noteworthy that *C. elegans* N2 RNAi efficiency is not the strongest in this assay. Especially, milder phenotypes than full mother sterility, such as deformed larvae, embryonic lethality or retarded development, were often observed using *C. elegans* N2; this is also the case with *C.* sp. 20 but not other sensitive species where the effect of actin RNAi was stronger (Figure 2 and Table S1). *C. elegans* N2 RNAi efficiency is thus in a range that appears highly sensitive to the concentration of dsRNAs experienced by the animals on the plates. This high sensitivity may explain the quantitative variability on different days of assays on N2 (e.g. Figure 2 and Table S1).

We note that the number of progeny in the negative controls at 23°C was consistently lower in species of the *Drosophilae* group compared to those of the *Elegans* super-group. The number of viable progeny, embryonic delay or arrest varied highly among parents. Lowered fecundity may be partly due to inbreeding depression in these obligate outcrossing species. Dead embryos were also observed in some species of the *Elegans* super-group, particularly those with a male-female mode of reproduction.

In order to determine the direction and pattern of change in sensitivity to ingested *Oti-actin* dsRNAs within the *Caenorhabditis* genus, we made use of a molecular phylogeny of the genus [17]. The pattern of sensitivity and insensitivity is complex when the phylogenetic relationships in the *Caenorhabditis* genus are taken into account. The state at the base of the tree is unclear. *C.* sp. 1 was not tested here but was previously shown to be sensitive to external *ama-1/pol-2* dsRNAs. A loss of sensitivity in the branch leading to *C. plicata* is the most parsimonious hypothesis, affecting both the response to ingested *Oti-actin* and *C. plicata ama-1/pol-2* dsRNAs. In the rest of the tree, some pattern emerges. Species in three clades are insensitive: 1) the current sister clade of *C. elegans*, i.e. eight species including *C. briggsae*, *C. remanei* and *C. brenneri* (except for a weak sensitivity in *C.* sp. 16); 2) the clade including *C.* sp. 17, *C.* sp. 18 and *C.* sp. 19; 3) the clade of five species including *C. angaria* and *C. drosophilae*. From the relationships among sensitive and insensitive species, the most parsimonious possibility is that these three clades have independently lost the ability to respond to ingested *Oti-actin* dsRNAs. However, gains in the branches leading to the clades 1) *C.* sp. 20; 2) *C.* sp. 6 and *C.* sp. 13; 3) *C.* sp. 7 and *C.* sp. 14; 4) *C. elegans*, and possibly 5) *C.* sp. 15 depending on its position are also possible, or a combination of gains and losses. In any case, a minimum of four character changes occurred in the *Caenorhabditis* genus, and of six when including the weak sensitivity of *C.* sp. 16 and intraspecific evolution in *C. elegans*.

In *C. elegans*, different wild isolates were previously shown to be variably sensitive to either germ line or somatic RNAi [9], [30]. The *C. elegans* N2 reference strain is sensitive in all tissues, albeit to different degrees, e.g. neurons are poorly responsive [31], [32]. By contrast, the *C. elegans* CB4856 wild isolate is insensitive to germ line RNAi [30]. The *C. elegans* JU1580 wild isolate was previously found to be insensitive to somatic RNAi (against muscle *Cel-unc-22* and GFP; [9], [30]) but sensitive to germ line RNAi (*Cel-pos-1*). We found that JU1580 is insensitive to *Oti-actin* dsRNAs (Figure 3 and Table S1) for all tested phenotypes: deformed larvae, dead embryos and sterile mothers (Table S1). This raises the possibility that the scored phenotypes after *Oti-actin* RNAi are solely the result of somatic inactivation of actin gene expression.

By contrast with the intraspecific variation in *C. elegans*, we find no variation within *C. briggsae*, nor with its close relatives: none of the three tested *C. briggsae* isolates was sensitive to external actin dsRNAs, nor were the most closely related species to *C. briggsae*, *C.* spp. 9 and 5 (Figure 3).

### Further tests of RNAi sensitivity in a subset of species

Concerning the species that are insensitive to ingested *Oti-actin* dsRNAs, we further sought to test whether (1) the external administration of dsRNAs or (2) the efficiency of RNAi against the *Oti-actin* gene (irrespective of its mode of administration) were defective. Previous work had shown that *C. briggsae*, *C. remanei* and *C. brenneri* do not respond to a variety of ingested dsRNAs yet are capable of responding to injected dsRNAs [5]. Among the new species in the same clade, we tested the hermaphroditic *C.* sp. 11 for its ability to respond to internally administered dsRNAs by injection of *Oti-actin* dsRNAs into the gonad. We found *C.* sp. 11 to be sensitive to these internally administered dsRNAs.This rules out a loss of sensitivity due to the evolution of actin gene sequences. It is thus most likely that the whole clade of eight species is defective in the entry of dsRNAs yet has maintained a response to internally administered dsRNAs, allowing for RNA interference studies using injection. In the case of *C. brenneri*, only injection into the gonad and not into the body cavity was previously found to be effective, suggesting a further inability for systemic spread among tissues in at least in this species [5].

Concerning species of the *Drosophilae* super-group that are defective in the reponse to external actin dsRNAs, Winston et al. [5] had shown that *C.* sp. 2 was capable of responding to injected *C.* sp. 2 *ama-1* dsRNAs. We focused on its sister species, *C. drosophilae*, which is of special interest because of its interesting ecology [33] and on the recently described *C. angaria* (formerly called *C.* sp. 3) [34]. Similar to the results in *C.* sp. 2, we found *C. drosophilae* to be insensitive to *Cdr-ama-1* dsRNAs administered by feeding, but sensitive (although not highly) to the corresponding *Cdr-ama-1* dsRNA fragment administered by injection into the gonad. Like *C. briggsae*, *C. drosophilae* thus appears to be defective in responding to external but not to internal dsRNAs. Alternatively, a dose effect is possible, assuming that injection delivers a higher dose of dsRNAs than the feeding method.

**Figure 4:**
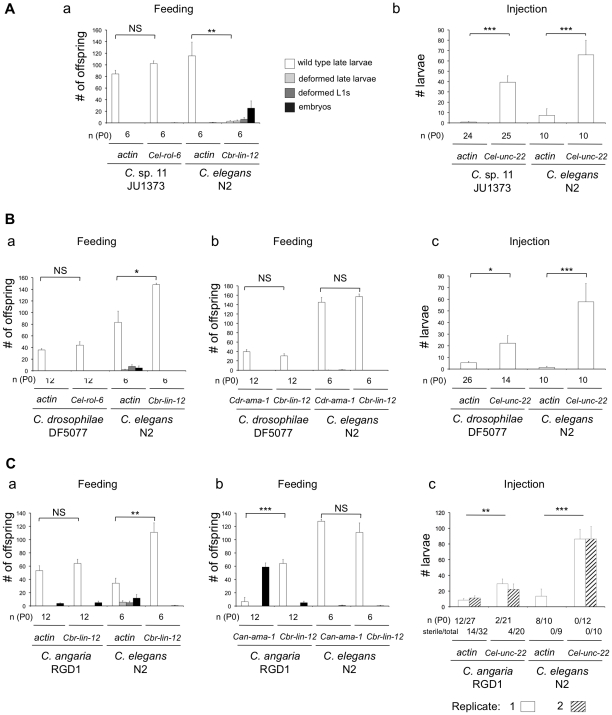
RNAi sensitivity of *C.* sp. 11, *C. drosophilae* and *C. angaria*: feeding versus injection. (**A**) *C.* sp. 11 JU1373 was found to be insensitive to *Oti-actin* RNAi using the feeding protocol (a), but sensitive to *Oti-actin* dsRNAs introduced by injection (b). (**B**) *C. drosophilae* DF5077 was found to be insensitive to RNAi by feeding using both *Oti-actin* (a) and *Cdr-ama-1* (b), but sensitive to *Oti-actin* dsRNAs introduced by injection (c). *C. drosophilae* has a smaller brood size than *C. elegans* in all experiments. (**C**) *C. angaria* RGD1 was found to be insensitive to RNAi by feeding using *Oti-actin* (a) but not *Can-ama-1* (b). (c) Compared to control animals injected with *Cel-unc-22* dsRNAs, *C. angaria* RGD1 animals injected with *actin* dsRNAs showed a significantly reduced number of larvae in their progeny. Injections of *actin* dsRNAs into *C. angaria* RGD1 animals were performed in two replicate experiments (shown in blue and red). The proportion of injected P0 that became fully sterile is reported below. Results for *C. elegans* are shown as positive control for the efficiency of the dsRNA treatment.

For *C. angaria*, we found that external administration of *Can-ama-1* dsRNAs by feeding - unlike that of *Oti-actin* dsRNAs - was highly effective. This result is surprising, because Winston et al. [5] saw no effect when soaking the animals in *Can-ama-1* dsRNAs. A dose effect, due to the dsRNA length or the soaking versus feeding mode of administration, could explain this discrepancy. Our own results showing a difference in sensitivity to ingested *Oti-actin* versus *Can-ama-1* dsRNAs may be explained in two ways: i) a better efficiency of, and/or higher dose sensitivity of the inactivation of *Can-ama-1* compared to that of *Oti-actin*; ii) a RNAi competency in only some tissues. Concerning the former hypothesis, upon inspection, the *Oti-actin* fragment sequence is less similar to the homologous *C. angaria* sequence (85% identity) than to those of *C. elegans* (90% identity), which may cause a partial silencing. Consistent with this hypothesis, *Oti-actin* dsRNAs can trigger a response when injected, yet with low efficiency (Figures 3 and 4). Concerning the latter hypothesis, *C. angaria* like *C. elegans* JU1580 ([10]; see above) may be specifically unable to raise a RNAi response in somatic tissues, rather than being sensitive to the mode of dsRNA administration. The inactivation of further genes in *C. angaria* will be necessary to distinguish between these alternatives. In any case, this species appears sensitive to some ingested dsRNAs.

### Production of *C. briggsae* and *C. remanei* strains that are sensitive to external dsRNAs

Species in the current sister clade to *C. elegans* were found to be insensitive to ingested dsRNAs. This clade includes species with sequenced genomes where RNAi would be a valuable tool [35] (www.wormbase.org). The RNA interference response could be activated when the dsRNAs were injected [5] (Figure 3). However, RNAi by feeding has several advantages for high-throughput screens [19] and for the screening of late developmental phenotypes by controlling the timing of dsRNA application. We thus set out to render some of the refractory *Caenorhabditis* species sensitive to dsRNAs administered by feeding, as a tool for further functional studies. This was previously achieved for *C. briggsae* by transforming a wild-type strain with *Cel-SID-2::GFP* [5]. The resulting *C. briggsae* line shows a Roller phenotype due to the *rol-6(d)/pRF4* transformation marker and displays intestinal GFP from the *Cel-SID-2::GFP* transgene. Each of these two features may render some other relevant phenotypes hard to score. In *C. briggsae*, we thus wished to obtain a non-Roller transgenic line that would help in the scoring of many phenotypes.

As in [5], we could “rescue” *C. briggsae* insensitivity to ingested dsRNAs with *Cel-sid-2*, using a simple PCR product of the *Cel-sid-2* gene instead of a fusion construct to GFP (see Materials and Methods). We first obtained an extra-chromosomal transgenic line in the *C. briggsae* AF16 wild background expressing *Cel-sid-2* and the *Cel-myo-2::DsRed2* marker. After irradiation and backcrosses to *C. briggsae* AF16, we derived two integrated transgenic strains, JU1018 and JU1076. Both strains were tested for their response to ingested dsRNAs (Table S2). JU1018 was found to be most sensitive to ingested *Oti-actin* dsRNAs, in fact more sensitive than the reference wild-type *C. elegans* strain N2; however, in the negative control, JU1018 displayed a somewhat low fertility. JU1076 was more fertile, but somewhat less sensitive to RNAi.

**Figure 5:**
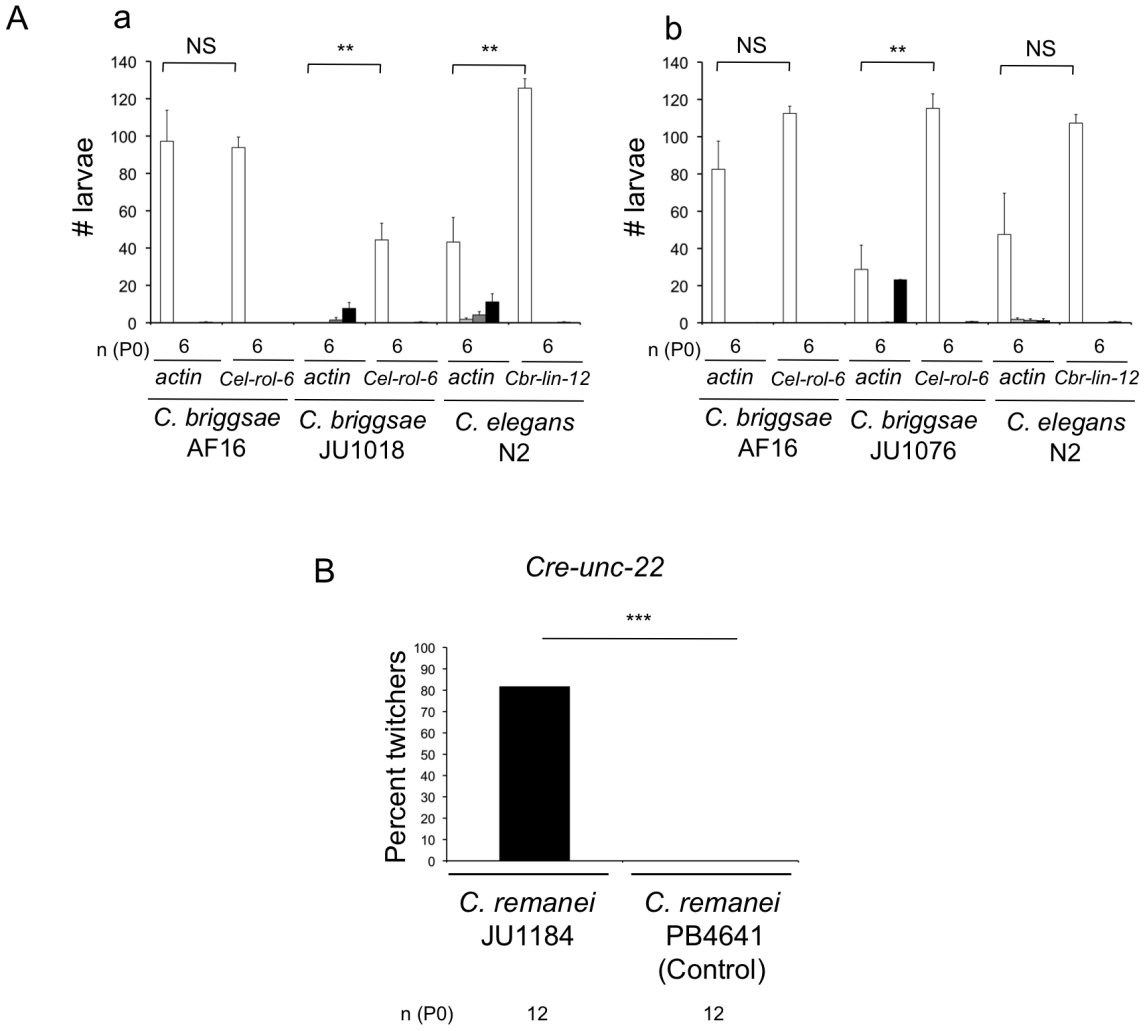
Complementing *C. briggsae* and *C. remanei* with *Cel-sid-2*. (**A**) Sensitivity to ingested *Oti-actin* RNAi of two *C. briggsae* integrated transgenic lines, JU1018 and JU1076, transformed with *Cel-sid-2* genomic DNA and a *myo-2::DsRed2* marker. Both lines are rendered sensitive to external actin dsRNA application, compared to the reference strain AF16. JU1018 has a lower brood size than the other lines. Statistical comparisons were made to the results of control experiments with *Cel-rol-6* or *Cbr-lin-12* dsRNA using a Mann-Whitney-Wilcoxon rank sum test on the number of normal larval progeny of each parent. Note that this test is very conservative and has low power. The significance of the difference is depicted as follows: (**NS**) non-significant, (*) 0.01<*p*<0.05, (**) 0.001<*p*<0.01, (***) *p*<0.001. Error bars indicate the standard error of the mean over individuals (n = 6–12, see Table S1 for details). (**B**) Sensitivity to *Cre-unc-22* RNAi by feeding of a *C. remanei* transgenic line, JU1184, transformed with *Cel-sid-2* genomic DNA and a *myo-2::DsRed2* marker. The transgenic strain *C. remanei* JU1184 displayed the characteristic twitching phenotype when *Cre-unc-22* dsRNAs were administered by feeding, whereas no twitcher was seen in the reference strain *C. remanei* PB4641. χ^2^ test: *p* = 10^−75^.

We next tried to similarly render *C. remanei* sensitive to ingested dsRNAs. We obtained the JU1184 strain by injection of *Cel-sid-2* and *Cel-myo-2::DsRed2* into the *C. remanei* PB4641 inbred strain, yielding the *mfEx34* transgene. This transgene is transmitted at a 100% frequency and may thus be a spontaneous integrant. This strain displayed a relatively low fertility that rendered the actin test difficult to interpret (since it is based on progeny number) (Table S2). We therefore cloned into the RNAi feeding vector a fragment of the endogenous *Cre-unc-22* gene, whose inactivation was predicted to produce a specific Twitcher phenotype [36]. Indeed, whereas we did not observe any Twitcher worms in the control conditions, over 80% of JU1184 progeny raised on bacteria expressing *Cre-unc-22* dsRNAs did twitch (Figure 5). We have thus obtained a *C. remanei* transgenic line that is sensitive to external dsRNAs and may be used for RNA interference experiments.

Using a similar protocol, we obtained *C.* sp. 11 transgenic animals carrying the *Cel-sid-2* gene. However, we failed to see a strong improvement in external RNAi efficiency when feeding three of these lines with *Oti-actin* dsRNA expressing bacteria (Table S2). The reasons for this failure of complementing *C.* sp. 11 with *Cel-sid-2* may include insufficient transgene expression or the occurrence of another defective step in the RNAi pathway of this species, for example systemic spread as in *C. brenneri* [5].

### Transgenesis in different *Caenorhabditis* species

To our knowledge, this is the first report of transgenesis of *C. remanei* and in the newly isolated selfing *C.* sp. 11. We further tested whether we could transform *Caenorhabditis* species using the injection protocol developed for *C. elegans*, which leads to the formation of extra-chromosomal arrays containing the injected sequences [22]. As transformation markers, we tried a number of *C. elegans*-based sequences able to induce dominant phenotypes, namely a Roller phenotype or GFP expression (Table 1).

Most tested *Caenorhabditis* species could be transformed (but see below); however, not all markers were expressed in all species. For example the *hsp-16-18::GFP* marker was not expressed after heat-shock in *C. brenneri* nor *C. angaria* (Table 1A, B, D). By contrast, the *sur-5::GFP* marker (wide somatic cell expression) or the *myo-2::GFP* marker (pharyngeal expression) could be successfully used in all tested species of the *Elegans* and *Drosophilae* supergroups, including *C. brenneri*, *C. angaria* and *C.* sp. 2. In addition to *C. remanei*, *C. brenneri, C. angaria* and *C. sp. 2* (Table 1), we could transform *C.* sp. 11 (Table S2), *C.* sp. 9 and *C.* sp. 5 using *myo-2::GFP* or *myo-2::DsRed2* (data not shown). The extra-chromosome could be visualized by Hoechst staining of oocytes in *C. angaria* (data not shown), showing that in this species of the *Drosophilae* supergroup as in *C. elegans*, transgenic lines were obtained by formation of an additional chromosome.

The most basally branching *C.* sp. 1 could not be transformed using *sur-5::GFP* nor *myo-2::GFP* markers. It is unclear whether transgenesis *per se* or the expression of the markers were unsuccessful. *C.* sp. 1 displays a smaller body size and has a single functional gonadal arm, which renders injections more difficult. In addition, few progeny was laid after injection. The absence of transformed progeny may be attributed to these complicating factors rather than the intrinsic inability of *C.* sp. 1 to form extra-chromosomes. In *C. plicata*, expression of the *sur-5::GFP* could be observed in the F1 generation after injection and the lack of transformants may simply reflect an insufficient number of injected animals (Table 1).

Concerning the efficiency of transformation, *C. elegans*, and to a lesser extent *C. briggsae*, were transformed with a higher frequency of success than other tested species of the *Elegans* and *Drosophilae* groups, such as *C. remanei*, *C. brenneri*, *C. angaria* and *C.* sp. 2 (Table 1). In these latter species, more animals need to be injected to yield the same number of transgenic strains.

Among the transgenic lines, we observed in *C. remanei* and *C. brenneri* several lines with 100% transmission of the marker, suggesting direct integration in the genome (Table 1 and the *Cel-sid-2*-expressing *C. remanei* strain mentioned above). The proportion of direct integration over extra-chromosome formation appears higher in these species, which is perhaps accounted for by the low efficiency of extra-chromosome formation.

We thus could transform most tested species of the *Caenorhabditis* genus, using *C. elegans* markers and protocols. This contrasts with the difficulty in transforming nematodes of other genera, such as *Oscheius tipulae* [37] or *Pristionchus pacificus* [38].

## Discussion

Here we investigated the ability of all *Caenorhabditis* species in culture to respond to ingested *Oti-actin* dsRNAs. We find that this character evolves rapidly in the genus, likely through several independent losses, acting at different steps of the response. We will review the situation in the relevant evolutionary groups successively (Figure 3).

In the clade including *C. briggsae* and *C. remanei*, the loss of the ability to respond to ingested *Oti-actin* dsRNAs corresponds to the apparent inability to respond to any external dsRNAs, yet with the ability to respond to internal dsRNAs. The response of these two species to ingested dsRNAs can be rescued by transgenesis with the *Cel-sid-2* gene. In the sister clade, *C. brenneri* was previously shown to have further lost the capacity to mount a systemic response to internal dsRNAs, when they were injected into the body cavity instead of the gonad [13]. The inability of *C.* sp. 11 to be rescued by *Cel-sid-2* for the response to ingested dsRNAs may be explained similarly, although this was not directly tested. In this clade, it is noteworthy that we observed a weak but significant response in *C.* sp. 16.

In some *C. elegans* isolates, such as JU1580, the inability to respond seems to concern dsRNAs matching somatically-expressed genes but not germ line-expressed genes. Variation in RNAi efficiency among tissues has been observed repeatedly in *C. elegans*, either in wild strains [13], [30] or in mutants [31], [32], [39]. It is likely that RNAi responses are affected by tissue-specific factors.

We did not investigate further the loss of sensitivity to *Oti-actin* dsRNAs in the *Elegans* supergroup clade that includes the recently isolated *Caenorhabditis* spp. 17–19.

In the *Drosophilae* supergroup, *C. drosophilae* and *C.* sp. 2 seem to have lost the ability to respond to all ingested dsRNAs, but are still capable of mounting an RNAi response, albeit weak, to injected dsRNAs. In the sister clade, *C. angaria* also does not respond to *Oti-actin* dsRNAs when ingested, and weakly so when injected. However, in *C. angaria*, this is not true for other dsRNAs such as *Can-ama-1*, either because of tissue specificity as in JU1580 or because of low efficiency and dosage effects.

What may drive such evolution in the response to dsRNAs? We will briefly review possibilities concerning the responses to i) internal and ii) external dsRNAs. First, the response to *internal* dsRNAs is used in transposon silencing [40], [41] and in defense against a natural virus in *C. elegans*, which was isolated from the JU1580 strain [13]. Transposons or pathogens may lead to variable selection pressures and to the observed rapid evolution of the RNAi machinery [15] - particularly in the germ line for transposons and in the intestine for viruses. Increase in sensitivity may thus act in defense against transposons or viruses. Decrease in sensitivity may occur when no viral pathogen nor transposon activity are present, either by simple mutational degradation or because of a cost of maintaining the active pathway. Alternatively, one can envisage that viruses may have positive fitness effects in some environments, for example by protecting infected intestinal cells from further infection by a more potent pathogen. Second, for the ability to respond to *ingested* dsRNAs, it is unclear what may have driven the evolution, but a clear possibility is the occurrence of dsRNAs from pathogens such as viruses in the external environment and the intestinal lumen. This may occur if the viral particles are partially degraded or imperfectly assembled, or if an infected cell is partially ruptured in the individual or its neighbors [5]. Alternatively, the necessary machinery for recognition and import of ingested dsRNAs, including the *sid-2* gene, may be physiologically required for quite distinct molecular activities, yet also in relation with the changing external environment.

In conclusion, we showed that the RNAi machinery was altered several times during *Caenorhabditis* evolution and in different subpathways affecting entry of dsRNAs, their systemic spread or the response of specific tissues. We further showed that *C. remanei* like *C. briggsae* could be complemented to respond to ingested dsRNAs by transformation with the *Cel-sid-2* gene. We thus provide a set of *C. briggsae* and *C. remanei* strains that can be used for external RNAi experiments. In addition, we showed that transgenesis by simple injection of DNA into the syncytial gonad can be performed in most *Caenorhabditis* species and that the commonly used *Cel-myo-2::GFP* and *Cel-sur-5::GFP* plasmids are suitable transformation markers in *Caenorhabditis* species.

## Supporting Information

Table S1 **Sensitivity of** ***Caenorhabditis*** **species to ingested** ***Oti-actin*** **dsRNAs.** The result of each experiment is displayed in a separate sheet, with the counts of different phenotypic classes indicated for each assayed plate on the left, and the mean and standard error over a treatment on the right. Results were analyzed below with a Mann-Whitney-Wilcoxon rank sum test on the number of wild-type larval progeny of each parent. This test is conservative, first because it is a rank test and second because it does not take into account other phenotype classes. We also tested the number of deformed larvae and dead embryos for low sensitivity species such as *C.* sp. 20. Note that we assayed more species for external RNAi using *Oti-actin* (reported in Figure 3), but did not quantify the results in the same manner.
(XLS)

Table S2 **Sensitivity to external dsRNAs of** ***Cel-sid-2*** **transgenic lines in** ***C. briggsae*** **,** ***C. remanei*** **and** ***C.*** **sp. 11.** The result of each experiment is displayed in a separate sheet, as in Table S1.
(XLS)

## References

[1] Fire A, Xu S, Montgomery MK, Kostas SA, Driver SE (1998). Potent and specific genetic interference by double-stranded RNA in *Caenorhabditis elegans*.. Nature.

[2] Tabara H, Grishok A, Mello CC (1998). RNAi in *C. elegans*: soaking in the genome sequence.. Science.

[3] Timmons L, Fire A (1998). Specific interference by ingested dsRNA.. Nature.

[4] Winston WM, Molodowitch C, Hunter CP (2000). Systemic RNAi in *C. elegans* requires the putative transmembrane protein SID-1.. Science.

[5] Winston W, Sutherlin M, Wright AJ, Feinberg EH, Hunter CP (2007). *Caenorhabditis elegans* SID-2 is required for environmental RNA interference.. Proc Natl Acad Sci USA.

[6] Louvet-Vallée S, Kolotuev I, Podbilewicz B, Félix M-A (2003). Control of vulval competence and centering in the nematode *Oscheius* sp. 1 CEW1.. Genetics.

[7] Pires da Silva A, Sommer R (2004). Conservation of the global sex determination gene *tra-1* in distantly related nematodes.. Genes Dev.

[8] Shannon A, Tyson T, Dix I, Boyd J, Burnell A (2008). Systemic RNAi mediated gene silencing in the anhydrobiotic nematode *Panagrolaimus superbus*.. BMC Mol Biol.

[9] Félix M-A (2008). RNA interference in nematodes and the chance that favored Sydney Brenner.. J Biol.

[10] Lu R, Maduro M, Li F, Li HW, Broitman-Maduro G (2005). Animal virus replication and RNAi-mediated antiviral silencing in *Caenorhabditis elegans*.. Nature.

[11] Schott DH, Cureton DK, Whelan SP, Hunter CP (2005). An antiviral role for the RNA interference machinery in *Caenorhabditis elegans*.. Proc Natl Acad Sci USA.

[12] Wilkins C, Dishongh R, Moore SC, Whitt MA, Chow M (2005). RNA interference is an antiviral defence mechanism in *Caenorhabditis elegans*.. Nature.

[13] Félix M-A, Ashe A, Piffaretti J, Wu G, Nuez I (2011). Natural and experimental infection of *Caenorhabditis* nematodes by novel viruses related to nodaviruses.. PLoS Biol.

[14] Obbard DJ, Jiggins FM, Halligan DL, Little TJ (2006). Natural selection drives extremely rapid evolution in antiviral RNAi genes.. Curr Biol.

[15] Thomas JH (2006). Adaptive evolution in two large families of ubiquitin-ligase adapters in nematodes and plants.. Genome Res.

[16] Dalzell JJ, McVeigh P, Warnock ND, Mitreva M, Bird DM (2011). RNAi effector diversity in nematodes.. PLoS Negl Trop Dis.

[17] Kiontke K, Félix M-A, Ailion M, Rockman MV, Braendle C (2011). A phylogeny and molecular barcodes for *Caenorhabditis*, with numerous new species from rotting fruits.. BMC Evol Biol.

[18] Delattre M (2001).

[19] Kamath RS, Fraser AG, Dong Y, Poulin G, Durbin R (2003). Systematic functional analysis of the *Caenorhabditis elegans* genome using RNAi.. Nature.

[20] Ahringer J., The *C. elegans* Research Community, ed. Reverse genetics.. http://www.wormbook.org/.

[21] Mello CC, Kramer JM, Stinchcomb D, Ambros V (1991). Efficient gene transfer in *C. elegans* after microinjection of DNA into germline cytoplasm: extrachromosomal maintenance and integration of transforming sequences.. EMBO J.

[22] Evans TC., The *C. elegans* Research Community, ed. Transformation and microinjection.. http://www.wormbook.org/doi/10.1895/1.108.1.

[23] Bessereau J-L, Wright A, Williams DC, Schuske K, Davis MW (2001). Mobilization of a *Drosophila* transposon in the *Caenorhabditis elegans* germ line.. Nature.

[24] Gu T, Orita S, Han M (1998). *Caenorhabditis elegans* SUR-5, a novel but conserved protein, negatively regulates LET-60 Ras activity during vulval induction.. Mol Cell Biol.

[25] Hsieh J, Liu J, Kostas SA, Chang C, Sternberg PW (1999). The RING-finger/B-box factor TAM-1 and a retinoblastoma-like protein LIN-35 modulate context-dependent gene silencing in *Caenorhabditis elegans*.. Genes Dev.

[26] Okkema PG, Harrison SW, Plunger V, Aryana A, Fire A (1993). Sequence requirements for myosin gene expression and regulation in *Caenorhabditis elegans*.. Genetics.

[27] Dichtel-Danjoy M-L, Félix M-A (2004). The two steps of vulval induction in *Oscheius tipulae* CEW1 recruit common regulators including a MEK kinase.. Dev Biol.

[28] Kiontke K, Barrière A, Kolotuev I, Podbilewicz B, Sommer RJ (2007). Trends, stasis and drift in the evolution of nematode vulva development.. Curr Biol.

[29] Velarde N, Gunsalus KC, P F (2007). Diverse roles of actin in *C. elegans* early embryogenesis.. BMC Dev Biol.

[30] Tijsterman M, Okihara KL, Thijssen K, Plasterl RHA (2002). PPW-1, a PAZ/PIWI protein required for efficient germline RNAi, is defective in a natural isolate of *C. elegans*.. Curr Biol.

[31] Simmer F, Tijsterman M, Parrish S, Koushika SP, Nonet ML (2002). Loss of the putative RNA-directed RNA polymerase RRF-3 makes *C. elegans* hypersensitive to RNAi.. Curr Biol.

[32] Kennedy S, Wang D, Ruvkun G (2004). A conserved siRNA-degrading RNase negatively regulates RNA interference in *C. elegans*.. Nature.

[33] Kiontke K (1997). Description of *Rhabditis (Caenorhabditis) drosophilae* n. sp. and *R. (C.) sonorae* n. sp. (Nematoda: Rhabditida) from saguaro cactus rot in Arizona.. Fundam appl Nematol.

[34] Sudhaus W, Kiontke K, Giblin-Davis RM (2010). Description of *Caenorhabditis angaria* n. sp. (Nematoda: Rhabditidae), an associate of sugarcane and palm weevils (Coleoptera: Curculionidae).. Nematology.

[35] Stein LD, Bao Z, Blasiar D, Blumenthal T, Brent MR (2003). The genome sequence of *Caenorhabditis briggsae*: a platform for comparative genomics.. PLoS Biol.

[36] Benian G, Kiff JE, Neckelmann N, Moerman D, Waterston RH (1989). Sequence of an unusually large protein implicated in regulation of myosin activity in *C. elegans*.. Nature.

[37] Félix M.-A., The *C. elegans* Research Community, ed. *Oscheius tipulae*.. http://www.wormbook.org/.

[38] Schlager B, Wang X, Braach G, Sommer RJ (2009). Molecular cloning of a dominant roller mutant and establishment of DNA-mediated transformation in the nematode *Pristionchus pacificus*.. Genesis.

[39] Zhuang JJ, Hunter CP (2011). Tissue specificity of *Caenorhabditis elegans* enhanced RNA interference mutants.. Genetics.

[40] Ketting RF, Haverkamp TH, van Luenen HG, Plasterk RH (1999). Mut-7 of *C. elegans*, required for transposon silencing and RNA interference, is a homolog of Werner syndrome helicase and RNaseD.. Cell.

[41] Sijen T, Plasterk RHA (2003). Transposon silencing in the *Caenorhabditis elegans* germ line by natural RNAi.. Nature.

